# Safety and outcomes of short-term use of peripheral vasoactive infusions in critically ill paediatric population in the emergency department

**DOI:** 10.1038/s41598-022-20510-2

**Published:** 2022-09-29

**Authors:** Y. Q. Yeong, J. M. F. Chan, J. K. Y. Chan, H. L. Huang, G. Y. Ong

**Affiliations:** 1 SingHealth Emergency Medicine Residency Program, Singapore Health Services, Singapore, Singapore; 2 KK Women’s and Children’s Hospital, Singapore, 100 Bukit Timah Road, Singapore, 229899 Singapore; 3 Duke-NUS Graduate Medical School, Singapore, 8 College Road, Singapore, 169857 Singapore

**Keywords:** Health care, Paediatrics, Paediatric research

## Abstract

Early restoration of oxygen delivery to end organs in paediatric patients experiencing shock states is critical to optimizing outcomes. However, obtaining central access in paediatric patients may be challenging in non-intensive care settings. There is limited literature on the use of peripheral vasoactive infusions in the initial resuscitation of paediatric patients in the emergency department. The aims of this study were to report the associated complications of peripheral vasoactive infusions and describe our local experience on its use. This was a single-centre, retrospective study on all paediatric patients who received peripheral vasoactive infusions at our paediatric emergency department from 2009 to 2016. 65 patients were included in this study. No patients had any local or regional complications. The mean patient age was 8.29 years old (± 5.99). The most frequent diagnosis was septic shock (45, 69.2%). Dopamine was the most used peripheral vasoactive agent (71.2%). The median time to central agents was 2 h (IQR 1–4). 16(24.2%) received multiple peripheral infusions. We reported no complications of peripheral vasoactive infusions. Its use could serve as a bridge till central access is obtained. Considerations on the use of multiple peripheral vasoactive infusions in the emergency department setting needs further research.

## Introduction

Paediatric shock carries a significant burden of disease. Particularly in septic shock, there is still a significant gap in mortality and morbidity outcomes in developed and developing countries^1^. In paediatric shock, timely administration of volume resuscitation and vasoactive infusions are crucial to restore organ perfusion and hence optimize mortality and morbidity outcomes^2–4^. There have been traditional concerns of local adverse reactions with vasoactive infusions administered via peripheral intravenous lines, with preference to administer them through central venous lines^5–19^. However, even in the hands of experienced teams, central venous lines are not easily obtained in the initial resuscitation of critically ill paediatric patients^3,4^. This is even more difficult in resource limited communities with limited access to tertiary paediatric care^1,16,17^. These factors could potentially lead to delay in the timely administration of vasoactive infusions.

There has been increasing recommendation for prompt administration of peripheral vasoactive infusions, especially in paediatric septic shock^2,4^. Recent studies of administration of peripheral vasoactive infusions demonstrated good safety profile, with local adverse reactions approximated at 2% for both adults and children^9^. Serious adverse events requiring medical or surgical interventions were infrequent^5–17^.

The aim of this study is to report the prevalence of local and regional complications of all children who were given vasoactive infusions via peripheral intravenous lines in the emergency department of a tertiary paediatric hospital, from the start of infusion to hospital discharge or death.

## Methods

This was a retrospective observational cohort study of all children who had received peripheral vasoactive infusions at the paediatric emergency department (PED) of KK Women’s and Children’s Hospital, Singapore, from June 2009 to May 2016.

### Setting

Our hospital is the only hospital dedicated to children’s and women’s health in Singapore, with a population of 5.7 million people. It has a bed capacity of 830 and its PED sees more than 130,000 paediatric patients (≤ 18 years old) annually.

There are pre-existing hospital protocols for patients receiving high risk infusates, which had been employed for the entire duration of this study. This includes patients receiving peripheral vasoactive infusions. These peripheral venous access sites are monitored for pain, swelling, erythema, leakage, and obstruction. This is done by the nursing team at timepoints of 0, 15, 30, 60, and 90 min from the point of line insertion, and hourly thereafter*.* Any signs of infiltration and/or extravasation is surfaced to the team senior doctor, with a prompt consideration for a referral to the Plastic Surgery team. All local adverse reactions are recorded in the medical and nursing records. Please refer to the Supplementary information for further details.

The study was approved by the Institutional Review Board of SingHealth (CIRB 2018/2256). The SingHealth Centralized Institutional Review Board had approved the waiver of informed consent for medical records review as this was a retrospective analysis of anonymised data, and the treatment conducted within the study was conducted according to existing clinical guidelines.

### Data collection

The study included paediatric patients who presented to the PED and had peripheral vasoactive infusions administered during the study period. Patients who did not survive to admission to the Intensive Care Unit (ICU) from the PED were excluded as we could not ascertain if there were any local or regional complications from the use of peripheral vasoactive infusions. Patients who received vasoactive infusions via central venous lines or had only bolus doses of intravenous vasoactive agents without any continuous infusion(s) in the PED were excluded.

The primary outcome was the presence of local and regional complications that arose from the use of vasoactive infusions through peripheral intravenous lines. These include local events such as extravasation, infiltration, and tissue necrosis. We defined a priori local and regional complications as extravasation of peripheral vasoactive infusions, tissue necrosis or limb ischaemia, from the time of initiation of the vasoactive infusion(s), to the point of hospital discharge or death. The secondary outcomes pertain to the patients’ clinical diagnoses, epidemiological data, and data on the administration of vasoactive infusions.

All patients who received vasoactive infusions in the PED based on our electronic and hardcopy medication charts and records in our study period were reviewed. To study our primary aim on the rates of local and regional complications associated with the use of peripheral vasoactive infusions, we actively sought and reviewed all patients’ PED and ICU records, which included but were not exclusive to all manual and electronic nursing charts for peripheral vascular access, high-risk medication infusion and monitoring charts, medical charts and problem lists in the ICU admission. We also actively searched for inpatient referrals and reviews by plastic, hand, orthopaedic or vascular surgeons during the period of intensive care admission for these patients. Active search for use of phentolamine in the electronic medication charts of all patients who received peripheral vasoactive infusions was done.

### Statistical analysis

Data was keyed into Microsoft Excel and analysed using IBM SPSS Statistics v25.0. Categorical variables were presented in frequencies and percentages. Test of skew and kurtosis were run for continuous variables. If this met Normal distribution, Mean (± standard deviation, SD) was used, otherwise Median (and interquartile range, IQR) was used.

### Ethics approval and consent to participate

The study was approved by our institution’s Institutional Review Board (CIRB 2018/2256). Electronic and hardcopy medical records were reviewed for this study.

## Results

**Figure 1:**
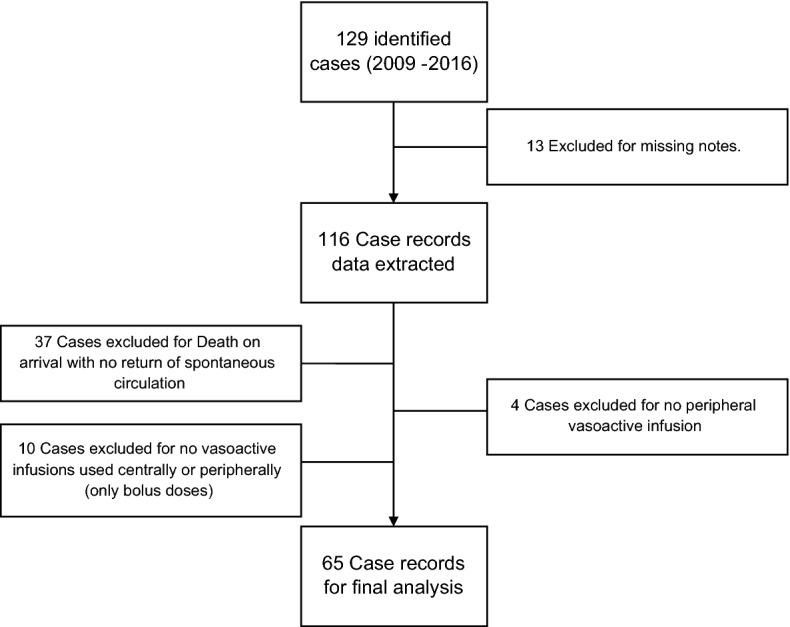
Strengthening the reporting of observational studies in epidemiology (STROBE) flowchart for the included patients of all patients identified with the use of vasoactive drugs in the PED.

A total of 129 cases were identified over the 8-year period examined. 13 cases were excluded for missing notes. 37 cases were excluded as the patient had died in the PED without return of spontaneous circulation. Further 18 cases were excluded as they did not receive vasoactive infusions but were only given boluses of vasoactive agents in the PED. The flowchart for the cases included for final analysis is illustrated in Fig. 1.

A total of 65 cases were included for final analysis. There were no local or regional complications due to peripheral vasoactive infusions on review. There were no medical records on the use of phentolamine, referrals to, or clinical notes by plastic surgery, orthopaedic or hand surgery teams for extravasation injuries due to peripheral vasoactive infusion.

The characteristics of the patients who were given peripheral vasoactive infusions are reported in Table 1.

**Table 1:** Epidemiology and clinical outcomes of paediatric patients who received peripheral vasoactive infusions in the PED.

Characteristics	
Age, years—Mean (SD)	8.29 (± 5.99)
Male (n, %)	35 (53.8%)
Diagnosis (n, %)	
Septic shock	45 (69.2%)
Myocarditis/Cardiomyopathy	6 (9.2%)
Submersion injury	4 (6.2%)
Cardiac arrest (respiratory causes)	3 (4.6%)
Cardiac arrest (out-of-hospital, unknown cause)	3 (4.6%)
Other	4 (6.2%)
Lowest recorded blood pressure in the emergency department (prior to peripheral VAIs^+^), mmHg	N = 47 (18 patients with cardiac arrest as below)
Mean SBP* (SD)	67.85 (± 16.45)
Mean MAP^#^ (SD)	48.63 (± 13.40)
Total number of patients who were in cardiopulmonary arrest (n, %)	18 (27.7%)
Out of hospital cardiopulmonary arrest (n, %)	16 (24.6%)
In hospital cardiopulmonary arrest (n, %)	2 (3.1%)
Initial Systolic blood pressure on arrival in ICU^^^ for patients on peripheral VAIs^+^, mmHg	n = 55 (10 missing)
Mean SBP* (SD)	96.0 (± 24.0)
Mean MAP^#^ (SD)	65.8 (± 22.8)
Median MAP^#^ (SD^)≠^	58.7 (52.7–72.3)
Local/Regional Complications due to peripheral VAIs^+^	
Extravasation	0
Tissue necrosis	0
Limb ischaemia	0
Duration of ICU stay (days)	3 (2–7)
Duration of hospital stay (days)	8 (5–19.0)
Outcome: survival to discharge	46 (70.8%)

^+^VAIs (Vasoactive infusions), * SBP (Systolic blood pressure), ^#^ MAP (Mean arterial pressure).

^^^ICU (Intensive care unit), ^≠^Median reported as not meeting test of skew/ kurtosis.

The mean age was 8.29 years old (± 5.99), and 35 (53.8%) were male. The most frequent primary diagnosis was septic shock (45 patients, 69.2%). There were 18 cases (27.7%) who had cardiopulmonary collapse, of which 16 were out-of-hospital cardiac arrest and 2 had cardiac arrest in the PED (in-hospital). While all included patients had hypotension in the emergency department, majority of these patients (60.7%) on peripheral vasoactive infusions had age-appropriate initial systolic blood pressures on arrival at the intensive care unit. 46 (70.8%) patients had an overall survival to discharge. There were 19 deaths in total, from sepsis (11), neurogenic shock (1), cardiorespiratory arrest (2), submersion (4), and trauma (1).

The peripheral venous access sites and characteristics of the vasoactive infusions used are summarised in Table 2. The most common site used for peripheral vasoactive infusion administration was the upper limb with 62 instances (51.5%). Most patients (70.8%) required 2 or more central vasoactive infusions subsequently. The median duration of peripheral vasoactive infusions initiated in the PED was 2.38 (QR 1.5–4.0) hours. The median time to fully transit to central vasoactive infusion was 2 h (IQR 1.0–4.0).

**Table 2:** Characteristics of peripheral access sites and peripheral vasoactive infusions used in the emergency department and subsequent intensive care management.

Characteristics	PED^x^-initiated Peripheral VAIs^+^ (N = 65)	Subsequent Central VAIs^+^ In the ICU^^^ (N = 63*)
Intravenous access sites (may be more than 1 site)		
Upper limbs		
Cubital fossa and above	12 (9.2%)	
Below elbow	55 (42.3%)	
Lower limbs		
Above ankle	12 (9.2%)	
Below ankle	6 (4.6%)	
Others		
Neck (external jugular)	8 (6.2%)	
No second site documented	37 (28.5%)	
Vasoactive infusion agents used (%^#^)		
Adrenaline (n, %)	23 (34.8%)	36 (55.4%)
Median infusion rate, mcg/kg/min (IQR)	0.1 (0.1–0.2)	0.17 (0.1–0.29)
Maximum infusion rate, mcg/kg/min	0.5	2.2
Noradrenaline (n, %)	6 (9.1%)	27 (41.5%)
Median infusion rate, mcg/kg/min (IQR)	0.1 (0.088–1.25)	0.15 (0.10–0.20)
Maximum infusion rate, mcg/kg/min	0.2	0.5
Dopamine (n, %)	47 (71.2%)	50 (76.9%)
Median infusion rate, mcg/kg/min (IQR)	10.0 (10.0–15.0)	10.0 (10.0–20.0)
Maximum infusion rate, mcg/kg/min	30	20
Dobutamine (n, %)	7 (10.6%)	8 (12.3%)
Median infusion rate, mcg/kg/min (IQR)	15 (10.0–30.0)	12.5 (10.0–27.5)
Maximum infusion rate, mcg/kg/min	30	30
Isoprenaline (n, %)	1 (1.5%)	0
Median infusion rate, mcg/kg/min (IQR)	0.3	
Maximum infusion rate, mcg/kg/min	0.3	
Milrinone (n, %)	0	4 (6.2%)
Median infusion rate, mcg/kg/min (IQR)		0.7 (0.25–0.7)
Maximum infusion rate, mcg/kg/min		0.7
Vasopressin (n, %)	0	9 (13.8%)
Median infusion rate, mcg/kg/min (IQR)		0.021 (0.014–0.050)
Maximum infusion rate, mcg/kg/min		0.06
Maximum number of concurrent VAIs^+^ used		
0	0	19 (29.2%)
1	49 (75.4%)	7 (10.8%)
2	14 (21.5%)	18 (27.7%)
3	2 (3.1%)	13 (20.0%)
4	0	4 (6.2%)
5	0	2 (3.1%)
Duration of VAIs^+^ (n = 63*)		
Median (IQR)	2.38 (1.5–4.0) hours	2 (1–4) days
Range (Minimum to Maximum)	0–48 h	1–17 days
Time to full transition of peripheral VAIs^+^ to central VAIs^+^, hours	N = 43 (22 incomplete records on transition time)	
Mean (SD)	2.6 (± 1.84)	
Range	0 to 8	

^x^PED (Paediatric emergency Department), ^+^VAIs (Vasoactive infusions), ^^^ICU (Intensive care unit), # percent for that VAI of total patient population, *2 patients with incomplete intensive care records.

We report the concurrent use of two or more vasoactive infusions in the PED in 16 cases (24.2%). The characteristics of patients who require multiple concurrent peripheral vasoactive infusions is described in Table 3. Of the 16 patients requiring multiple concurrent peripheral vasoactive infusions, we did not report any adverse effects. 9 (56%) of these 16 patients did not survive to discharge. 8 of these patients had septic shock, 7 had cardiac arrest, and 1 had myocarditis.

**Table 3:** Characteristics of patients requiring 2 or more concurrent peripheral vasoactive infusions.

Types of peripheral ≥ 2 VAIs^+^ running concurrently (n = 16, 24.4%)
**Combinations of 2 VAIs**^**+**^** running concurrently n = 14 (n,%)**
** Drug median infusion rate, mcg/kg/min (IQR)**
Adrenaline + Dopamine n = 9 (64.3%)
Adrenaline 0.2 (0.1–0.225)
Dopamine 10.0 (10.0–20.0)
Adrenaline + Noradrenaline n = 2 (14.3%)
Adrenaline 0.2 (0.2–0.2)
Noradrenaline 0.2 (0.2–0.2)
Adrenaline + Dobutamine n = 1 (7.1%)
Adrenaline 0.08*
Dobutamine 10*
Dopamine + Noradrenaline n = 1 (7.1%)
Dopamine 10.0*
Noradrenaline 0.1*
Dobutamine + Isoprenaline n = 1 (7.1%)
Dobutamine 30.0*
Isoprenaline 0.3*
**Combinations of 3 VAIs**^**+**^** running concurrently n = 2 (n,%)**
** Drug median infusion rate, mcg/kg/min (IQR)**
Adrenaline + Noradrenaline + Dopamine (n = 2)
Adrenaline 0.1 (0.1–0.1)
Dopamine 20 (10–20)
Noradrenaline 0.1 (0.1–0.1)

^+^VAIs (Vasoactive infusions), *no median (IQR) reported if n = 1.

## Discussion

In shock, prompt commencement of vasoactive infusions is crucial to improve haemodynamic function and restore end-organ perfusion^2–4^. Adherence to this in the first hour of septic shock has been associated with improved hospital mortality outcomes^3^.

Our case series and prior paediatric papers similarly suggested that the short-term use of peripheral vasoactive infusions was safe with low rates of minor adverse local or regional complications in critically ill infants and children^5,6,10–16^. A recent 2021 systematic review and meta-analysis that looked at both the paediatric and adult population found low risk of serious adverse events with peripheral vasoactive infusions^9^. However, these studies were predominantly in the intensive care and transport settings^5,6,10–16^. Emergency departments provide initial resuscitation and stabilisation of critically ill paediatric patients. However, many high-volume EDs (including our PED, which sees 350–500 paediatric patients a day), may not have ICU-level resources for longitudinal critical care such as uninterrupted and dedicated nursing care, and continuous invasive haemodynamic monitoring readily available^16,20^.Thus, setting-specific paediatric studies on the use of peripheral vasoactive infusions are important. To our knowledge, we know of only one other recent paediatric case series (49 patients) that studies the safety of peripheral vasoactive infusions exclusively in the setting of an emergency department^10^.

Generally, our paediatric population was older than reported in most other papers^5,6,10–16^. In our case series, we found that the median time to complete transition from peripheral to central vasoactive infusion was 2 h (IQR 1.0–4.0) as reported in Table 2. Most of these patients needed further haemodynamic support with multiple vasoactive infusions. The use of peripheral vasoactive infusions could allow for a more uninterrupted haemodynamic support with earlier commencement of vasoactive infusion(s) while obtaining and confirming the placement of central venous access, if clinically needed.

Interestingly we report the concurrent use of multiple (up to 3) peripheral vasoactive infusions. These were uncommon except in septic shock (8 patients) and cardiac arrest (7 patients) with hypotension following return-of-spontaneous circulation as presented in Table 3. There is little published paediatric literature on this, especially in the emergency department setting. While we reported no local or regional adverse reactions on the use of multiple peripheral vasoactive infusions, the very small numbers significantly limit any definitive conclusions that can be derived from this subpopulation from our case series. Further studies are required to evaluate the clinical utility and role of short-term concurrent use of multiple peripheral vasoactive infusions.

Our study’s findings on the safe use of peripheral vasoactive agents in the emergency department were likely contributed by our institutional practice of standardised vasoactive agent dilution for peripheral vasoactive infusions, protocols for their administration and close monitoring, and planned responses to local or regional complications. A copy of our institution’s monitoring protocol for peripheral vasoactive infusions (and other high risk infusates), and dilution guidelines for vasoactive infusions are included as supplementary information. We feel that it is important that we highlight that the generalisability of our study should be limited to paediatric emergency departments with a similar set-up. Transition to a longer-term central venous line should be done as soon as practicable, if clinically needed^10,17^.

The main limitations of our study were the retrospective nature of our study, small numbers, and the limited external validity of our findings due to the local practice of a single centre.

While we have complete data for most of our fields, there were 22 records (51%) where the time to full transition from emergency department-initiated peripheral vasoactive to central venous infusions were not clearly documented.

Lastly, we defined our study population with septic shock from ICU and hospital summaries. We did not interrogate the patients’ laboratory and other investigations or evaluate for organ dysfunction scoring systems (such as the Pediatric Logistic Organ Dysfunction-2 or Pediatric Sequential Organ Failure Assessment)^21–23^.

## Conclusion

This case series of paediatric patients receiving peripherally administered vasoactive infusions in the paediatric emergency department add to the growing body of evidence that local and regional complications are uncommon. Peripheral vasoactive infusions allow for early commencement of vasoactive infusions to achieve haemodynamic stability and improve organ perfusion. This serves as an important bridge until central access is obtained in critically ill paediatric patients with refractory haemodynamic instability. Considerations on the use of multiple peripheral vasoactive infusions in the emergency department setting need further research.

## Supplementary Information

Supplementary Information.

## Data Availability

The datasets used and/or analysed during the current study are available from the corresponding author on reasonable request.

## Abbreviations

ICU: Intensive Care Unit
IQR: Interquartile range
PED: Paediatric Emergency Department
SD: Standard deviation
VAI: Vasoactive infusion
