# Supplementary material for: Safety and outcomes of short-term use of peripheral vasoactive infusions in critically ill paediatric population in the emergency department
Source: Sci Rep. 2022 Sep 29;12:16340. doi: 10.1038/s41598-022-20510-2 (PMC9523065; doi:10.1038/s41598-022-20510-2)
Supplement: Supplementary file 1 — Supplementary Information. [file 41598_2022_20510_MOESM1_ESM.docx]

**Supplementary information**

**Appendix 1: Monitoring protocol for vasoactive infusions**

**
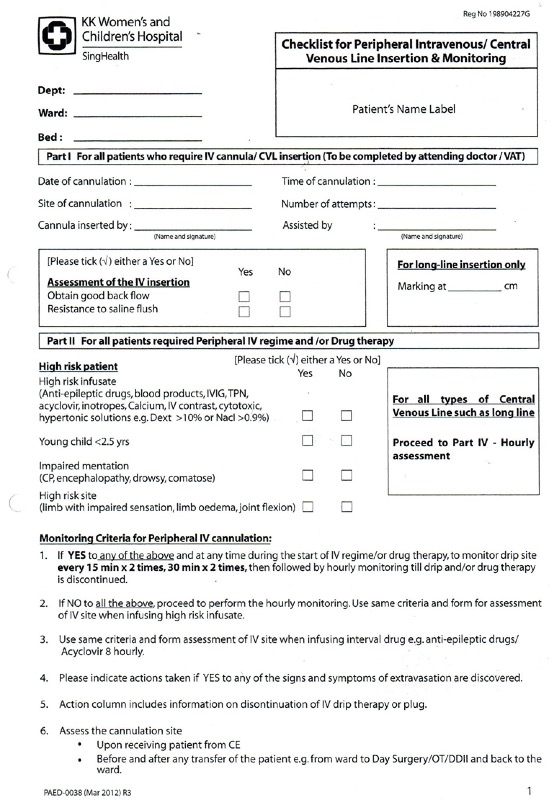
**


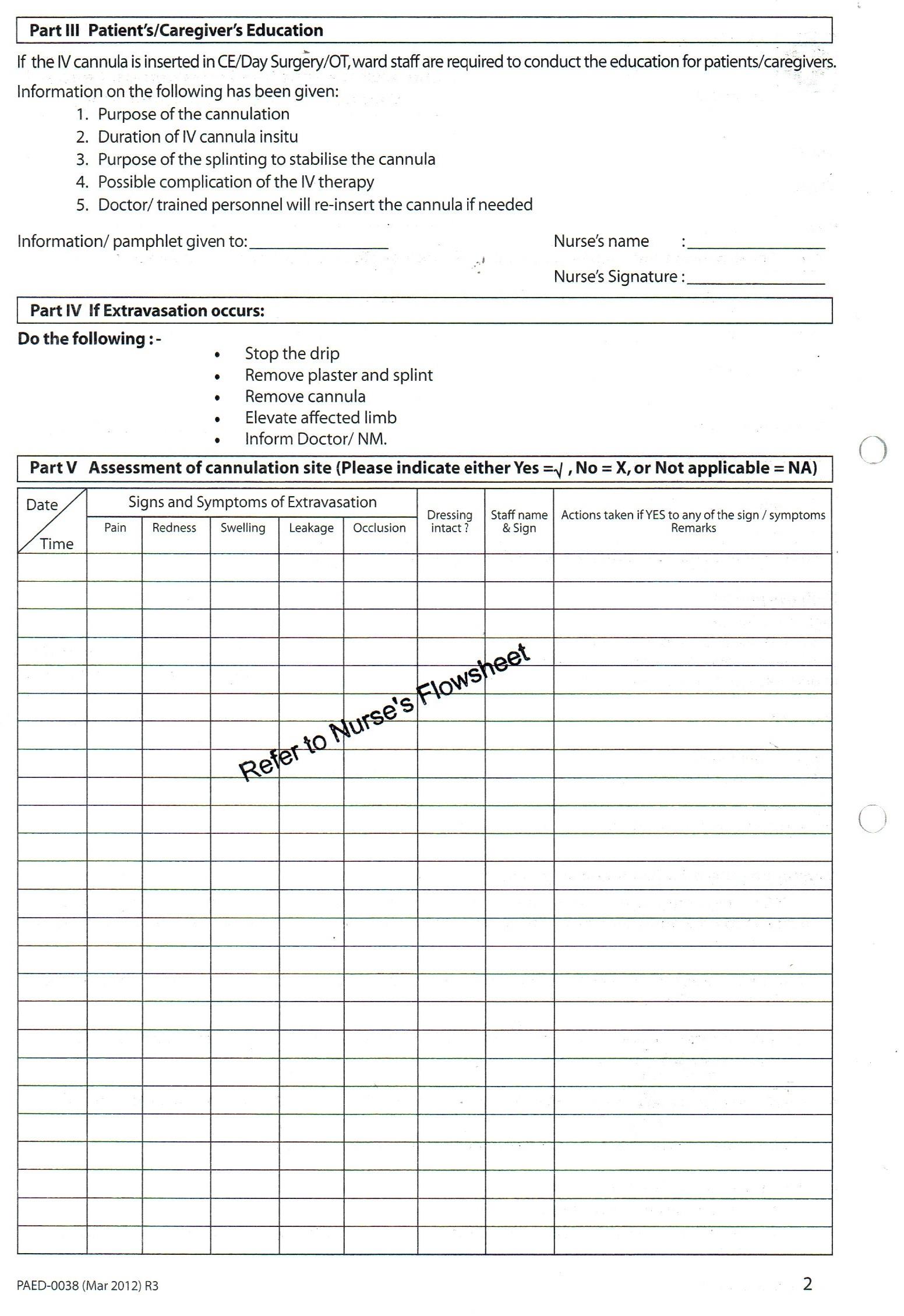


**Appendix 2: Dilution Guidelines for peripheral vasoactive infusion**

| **Peripheral vasoactive agent** | **Concentration** |
| --- | --- |
| Dopamine | 300 mcg/kg/ml |
| Dobutamine | 300 mcg/kg/ml |
| Adrenaline | 3 mcg/kg/ml |
| Noradrenaline | 3 mcg/kg/ml |
